# Supplementary material for: Mitochondrial fusion supports increased oxidative phosphorylation during cell proliferation
Source: eLife. 2019 Jan 29;8:e41351. doi: 10.7554/eLife.41351 (PMC6351101; doi:10.7554/eLife.41351)
Supplement: Figure 1—source data 1. — Data are presented as mean ±SEM (n = 4). [file elife-41351-fig1-data1.pptx]

## Slide 1
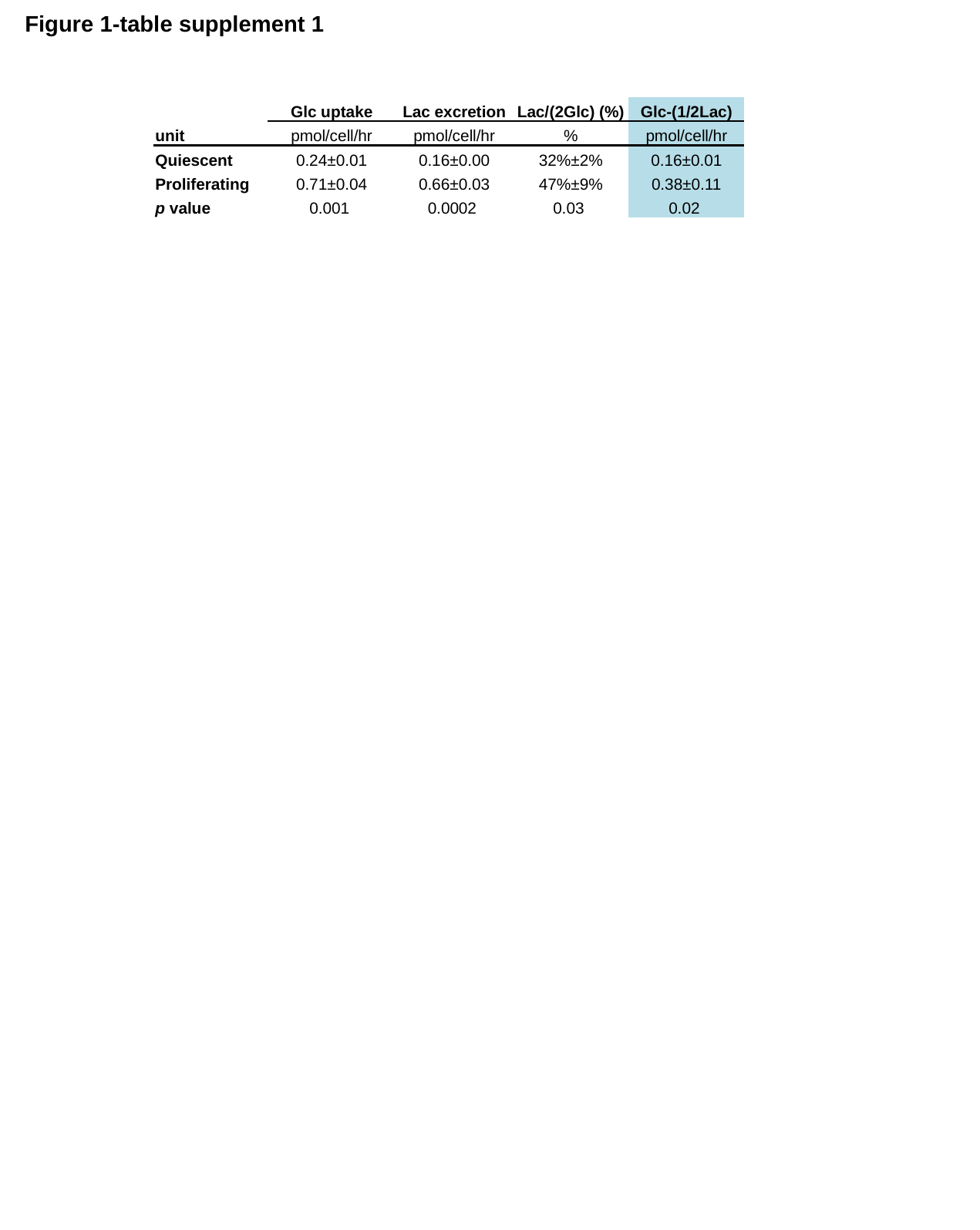

Figure 1-table supplement 1
| | Glc uptake | Lac excretion | Lac/(2Glc) (%) | Glc-(1/2Lac) |
| --- | --- | --- | --- | --- |
| unit | pmol/cell/hr | pmol/cell/hr | % | pmol/cell/hr |
| Quiescent | 0.24±0.01 | 0.16±0.00 | 32%±2% | 0.16±0.01 |
| Proliferating | 0.71±0.04 | 0.66±0.03 | 47%±9% | 0.38±0.11 |
| p value | 0.001 | 0.0002 | 0.03 | 0.02 |
