# Supplementary material for: Mitochondrial fusion supports increased oxidative phosphorylation during cell proliferation
Source: eLife. 2019 Jan 29;8:e41351. doi: 10.7554/eLife.41351 (PMC6351101; doi:10.7554/eLife.41351)
Supplement: Figure 1—source data 2. — Data are presented as mean ±SEM (n = 3). [file elife-41351-fig1-data2.pptx]

## Slide 1
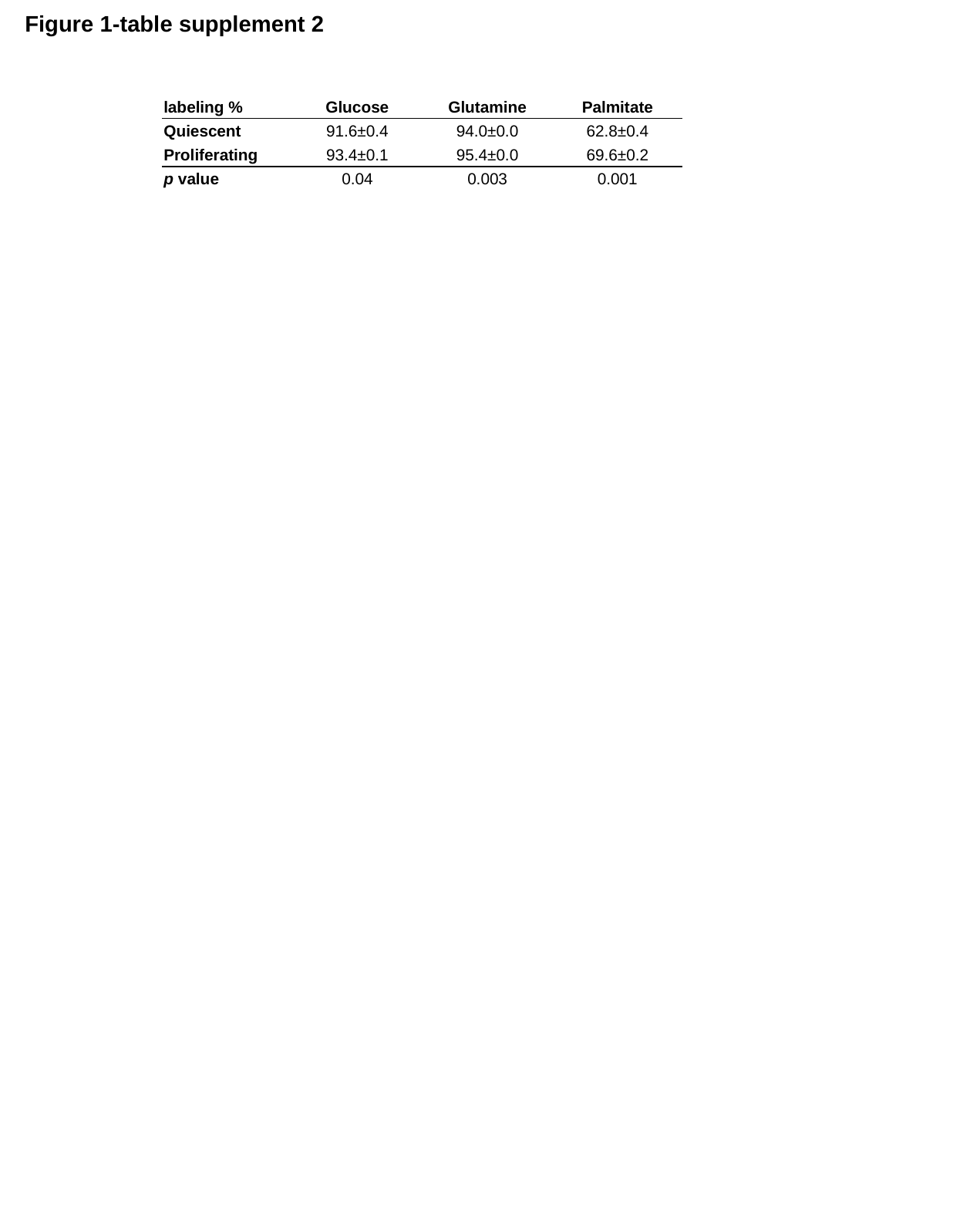

Figure 1-table supplement 2
| labeling % | Glucose | Glutamine | Palmitate |
| --- | --- | --- | --- |
| Quiescent | 91.6±0.4 | 94.0±0.0 | 62.8±0.4 |
| Proliferating | 93.4±0.1 | 95.4±0.0 | 69.6±0.2 |
| p value | 0.04 | 0.003 | 0.001 |
