# Supplementary material for: Mitochondrial fusion supports increased oxidative phosphorylation during cell proliferation
Source: eLife. 2019 Jan 29;8:e41351. doi: 10.7554/eLife.41351 (PMC6351101; doi:10.7554/eLife.41351)
Supplement: Figure 3—source data 1. [file elife-41351-fig3-data1.docx]

**Appendix S1.** Sequences for DsiRNA, siRNA resistant Mfn2^res^.

**The DsiRNA sequence for siRNA #1 (targeting CDS):**

5’- GCUAUGACCUGAAUUGUGACAAGCT-3’

3’ - GUCGAUACUGGACUUAACACUGUUCGA-5’

**The DsiRNA sequence for siRNA #2 (targeting CDS):**

5’- AUACGCCAGUGAGAAGCUACAGCTC-3’

3’ - CUUAUGCGGUCACUCUUCGAUGUCGAG-5’

**The DsiRNA sequence for siRNA #3 (targeting 3’UTR):**

5’- GAAAAAUUCAGGAUACUUCUGAAAT-3’

3’ - GUCUUUUUAAGUCCUAUGAAGACUUUA-5’

**The cDNA sequence for siRNA resistant Mfn2^res^:**

ATGTCCCTGCTCTTTTCTCGATGCAACTCCATCGTCACCGTCAAGAAGGATAAGCGACACATGGCTGAAGTGAATGCTTCCCCTCTCAAGCACTTTGTCACTGCCAAGAAAAAGATCAATGGAATCTTTGAGCAGCTGGGGGCCTACATCCAAGAGAGCGCCAGCTTCCTTGAAGACACCCACAGGAACACAGAACTGGACCCGGTTACCACGGAAGAGCAGGTCCTGGACGTCAAAGGGTACCTGTCCAAGGTCAGGGGTATCAGCGAAGTGCTGGCCAGGCGGCACATGAAGGTGGCTTTTTTTGGCCGGACGAGCAATGGGAAGAGCACCGTGATCAATGCCATGCTCTGGGACAAAGTTCTGCCATCTGGGATTGGTCATACCACCAATTGCTTCCTGCGGGTTGGGGGCACAGATGGCCATGAGGCCTTCCTCCTCACAGAGGGCTCAGAAGAGAAGAAGAGTGTCAAGACTGTGAACCAACTGGCCCATGCCCTCCATCAGGACGAGCAGTTGCATGCAGGCAGCATGGTGAGTGTGATGTGGCCCAACTCCAAGTGTCCGCTCCTGAAGGATGACCTCGTGCTGATGGACAGCCCTGGGATCGATGTTACCACGGAGCTGGACAGCTGGATTGATAAGTTTTGCCTGGATGCTGATGTGTTTGTGCTGGTGGCCAACTCAGAGTCCACGCTGATGCAGACGGAGAAGCAGTTCTTCCACAAAGTGAGTGAACGTCTCTCCCGGCCCAACATCTTCATCCTGAACAACCGCTGGGATGCGTCTGCCTCGGAGCCTGAGTACATGGAGGAGGTGCGGCGGCAGCACATGGAGCGCTGCACCAGCTTTCTGGTGGATGAGCTGGGCGTGGTGGATCGAGCTCAGGCTGGGGACCGGATCTTCTTCGTGTCTGCCAAGGAGGTTCTCAGCGCCAGGGTCCAGAAAGCCCAGGGCATGCCAGAAGGAGGCGGCGCTCTCGCAGAAGGTTTTCAAGTGAGGATGTTTGAGTTTCAGAATTTCGAGAGGCAGTTTGAGGAGTGCATTTCCCAGTCTGCAGTAAAGACCAAATTTGAGCAGCACACAGTCCGGGCCAAGCAGATTGCAGAGGCCGTTCGTCTCATCATGGATTCCCTGCACATCGCAGCTCAGGAGCAGCGGGTTTATTGCCTAGAAATGCGGGAAGAGCGGCAAGACCGGCTGAGGTTTATTGACAAGCAGCTGGAGCTCCTGGCTCAAGACTACAAGCTGCGAATTAAGCAGATTACGGAGGAAGTGGAAAGGCAGGTGTCCACAGCCATGGCTGAAGAGATCAGGCGCCTCTCTGTGCTAGTTGACGAGTACCAGATGGACTTCCACCCATCCCCAGTTGTCCTCAAGGTTTATAAGAACGAGCTGCACCGCCATATAGAGGAAGGTCTGGGCCGGAACCTGTCTGACCGCTGCTCCACTGCCATTGCCAGTTCACTGCAGACTATGCAGCAGGACATGATAGACGGCTTGAAGCCCCTTCTTCCTGTATCTATGCGGAATCAGATAGACATGCTGGTCCCTCGACAGTGTTTCTCCCTCTCTTACGATCTCAACTGCGATAAATTGTGTGCTGACTTTCAGGAGGACATCGAGTTCCACTTCTCCCTTGGATGGACTATGCTAGTGAACAGGTTCCTGGGCCCCAAGAATAGCCGCCGGGCCTTGCTAGGCTACAGTGATCAGGTTCAGCGTCCTCTCCCTCTGACACCTGCCAACCCCAGCATGCCCCCCTTGCCACAGAGCTCCCTCACCCAGGAGGAGCTCATGGTCTCCATGGTTACTGGCCTGGCCTCTCTGACGTCTAGGACCTCCATGGGCATTCTTGTGGTCGGAGGAGTGGTGTGGAAGGCAGTGGGCTGGAGACTCATCGCCCTCTCCTTTGGACTGTATGGCCTCCTGTACGTCTATGAGCGACTGACCTGGACCACCAAAGCCAAAGAGAGGGCCTTCAAGCGCCAGTTTGTGGAGTATGCTTCCGAAAAATTGCAATTGATCATCAGTTACACCGGCTCTAACTGCAGCCACCAAGTCCAGCAGGAATTGTCTGGGACATTTGCTCATCTGTGCCAGCAAGTTGACATCACCCGAGATAATCTGGAGCAGGAAATTGCTGCCATGAACAAGAAAGTCGAGGCTCTGGATTCACTTCAGAGCAGAGCCAAACTGCTCAGGAATAAAGCTGGCTGGTTGGACAGCGAACTCAACATGTTCACACACCAGTACCTGCAGCCCAGCAGATAG
